# Prevalence and Pathogenicity of WU and KI Polyomaviruses in Children, the Netherlands

**DOI:** 10.3201/eid1411.080464

**Published:** 2008-11

**Authors:** Marieke M. van der Zalm, John W. A. Rossen, Bart E. van Ewijk, Berry Wilbrink, Petra C.H.M. van Esch, Tom F.W. Wolfs, Cornelis K. van der Ent

**Affiliations:** Wilhelmina Children’s Hospital at University Medical Center Utrecht, Utrecht, the Netherlands (M.M. van der Zalm, B.E. van Ewijk, T.F.W. Wolfs, C.K. van der Ent); St. Elisabeth Hospital, Tilburg, the Netherlands (J.W.A. Rossen, P.C.H.M. van Esch); National Institute of Public Health and the Environment, Bilthoven, the Netherlands (B. Wilbrink)

**Keywords:** respiratory tract infections, viruses, child, preschool, dispatch

## Abstract

A longitudinal study in 2004 and 2005 detected polyomaviruses WU and KI in 44% and 17% of children with and without respiratory symptoms, respectively, in the Netherlands. In some children both viruses were detected for long periods. In several symptomatic children no other respiratory pathogen was detected.

High-throughput sequencing techniques have revealed 2 new polyomaviruses called the WU virus (WUPyV) (*1*) and KI virus (KIPyV) (*2*). WUPyV and KIPyV have been reported in respiratory samples of uncontrolled studies of small groups of hospitalized patients (*1*–*5*). However, the clinical relevance of these viruses in humans is unclear because data are lacking on these viruses in otherwise healthy persons outside a hospital setting (*6*–*8*). Whether these newly identified viruses also occur in healthy children and whether they should be seen as causative agents for clinical respiratory disease are not known. This study determined the prevalence of WUPyV and KIPyV in young children in the Netherlands with and without clinical respiratory symptoms.

## The Study

During a 6-month period (November 2004–April 2005), we performed a systematic survey on WUPyV and KIPyV and closely monitored respiratory symptoms in a prospective longitudinal cohort of 18 young children (<1–7 years of age, mean age 3.4 years from throughout the Netherlands). The study coordinator contacted parents twice a week (by telephone or email) to ask about the presence of any signs or symptoms of respiratory tract illness. Respiratory signs and symptoms were defined as coryza (rhinorrhea or nasal congestion), sore throat, ear ache with or without ear discharge, cough, sputum production or dyspnea, in the presence or absence of fever (>38°C). Every 2 weeks, samples for virus detection were collected regardless of any respiratory symptoms. A sample was defined as asymptomatic if the child had no respiratory symptoms 1 full week before and 1 full week after sampling. A sample was defined as symptomatic if the child had any respiratory symptoms 1 week before and 1 week after sampling. Parents collected the samples for virus detection by rubbing 1 nostril and posterior oropharynx of their child with separate cotton-tipped swabs. Feasibility of virus sampling by the parents has been described earlier (*9*). The study was approved by the local medical ethics committee, and the parents gave written informed consent.

**Figure 1:**
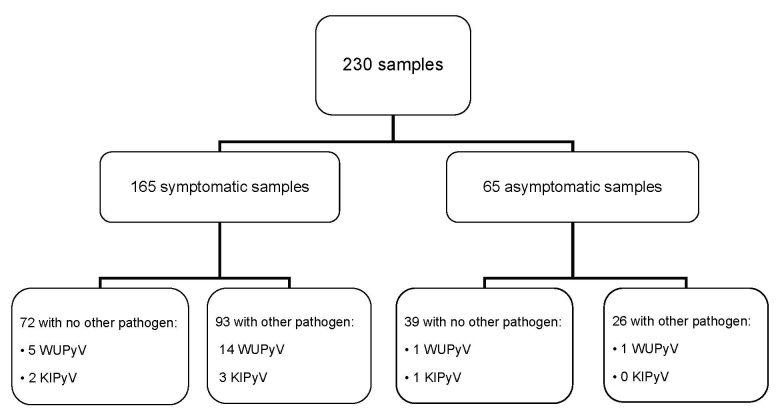
Flow chart of the respiratory samples taken in the study. Samples were collected during November 2004–April 2005, throughout the Netherlands. Samples were taken during symptomatic and asymptomatic episodes. Results show WU polyomavirus (WUPyV )and KI polyomavirus (KIPyV) detections in samples simultaneously negative for other respiratory pathogens and in samples in which >1 other respiratory pathogen(s) were detected.

In total 230 samples of symptomatic and asymptomatic samples were collected and tested for WUPyV and KIPyV by real-time PCR using an ABI 7500 System (Applied Biosystems, Foster City, CA, USA) (*10*) and for other respiratory pathogens by PCR (*11*) (Figure 1). In 119 samples (52%), the following nonpolyomavirus respiratory pathogens were detected: rhinovirus (32%); enterovirus (3%); respiratory syncytial viruses A and B (2%); coronaviruses OC43, 229E, and NL63 (17%); influenza viruses A and B (1%); human metapneumovirus (1%); adenovirus (<1%); *Mycoplasma pneumoniae* (3%); and *Chlamydophila pneumoniae* (5%).

WUPyV was found in 21 (9%) of 230 samples from 8 (44%) of 18 children (Figure 1). In 5 episodes WUPyV was the only pathogen detected and might therefore have been responsible for the observed respiratory symptoms. KIPyV was found in 6 (3%) of 230 samples in 3 (17%) of 18 children. In 2 symptomatic samples positive for KIPyV, no other pathogens were detected.

**Figure 2:**
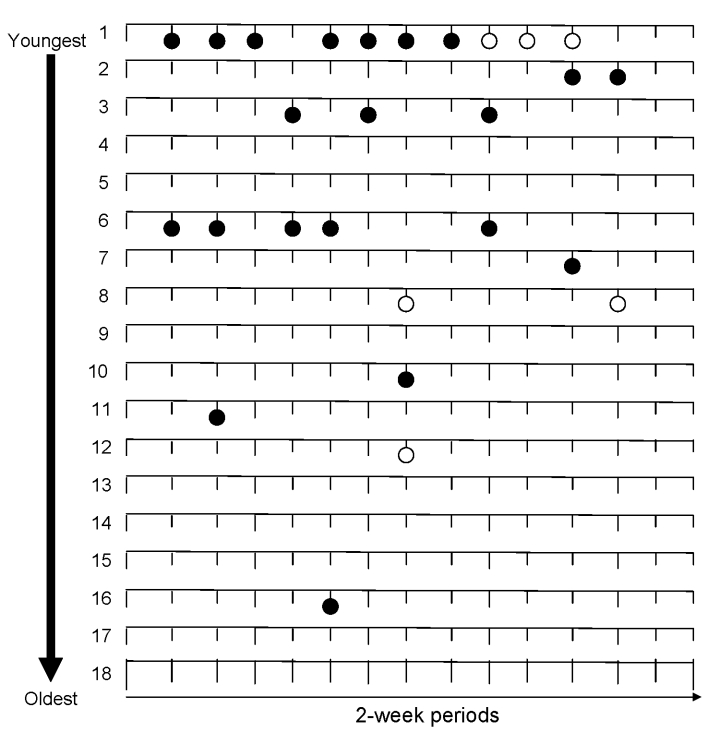
Timelines of WU polyomavirus (WUPyV ) and KI polyomavirus (KIPyV) in 2-week samples, taken regardless of symptoms. Samples were collected during November 2004–April 2005, throughout the Netherlands. Each line represents a child in order of increasing age (patients 1–18, aged <1–7 years); the time between 2 vertical lines accounts for ≈2 weeks. The solid symbols are WUPyV infections; the open symbols are KIPyV infections.

To track possible reinfections and/or prolonged infections of WUPyV and KIPyV during the observation period, we constructed Figure 2. For pediatric patients 1, 2, 3, and 6, WUPyV was found in >1 positive sample. In addition, prolonged presence of WUPyV (in >2 successive samples, equal to >2 weeks) was detected in 3 children (1, 2, and 6). The youngest child of the group (patient 1) had 2 periods of a prolonged infection with WUPyV (1 period of 3 successive positive samples and 1 period with 4 positive samples) as well as 3 successive KIPyV-positive samples. In the other children, KIPyV positivity was limited to 1 period.

Most infections with WUPyV and KIPyV were seen in the youngest children; 95% of the WUPyV infections and 83% of the KIPyV infections were seen in children <4 years of age (patients 1–10). For the children with a WUPyV infection, the median age was 2.6 years (interquartile range 0.7–3.9); for a KIPyV infection the median age was 3.0 years (interquartile range 0.4–4.0) (Figure 2)

## Conclusions

This unique prospective longitudinal cohort study shows a high occurrence of WUPyV and KIPyV in children. WUPyV and KIPyV were repeatedly observed as the only detectable pathogen in children with respiratory symptoms, which may suggest that both viruses have pathogenic potential. In addition, younger age was associated with a higher occurrence of WUPyV and KIPyV infections.

Our overall percentages of WUPyV and KIPyV are somewhat higher than percentages reported in most other studies (*1*–*8*). This finding may partially be explained by the fact that samples were collected during the respiratory disease season. In addition, our study had a longitudinal design, and, therefore, our prevalences cannot be compared with those of other studies. In our study WUPyV was the third most prevalent pathogen (9%) after rhinoviruses (32%) and coronaviruses (17%). KIPyV was the fifth most prevalent pathogen (3%), comparable with enteroviruses.

The pathogenic role of WUPyV and KIPyV in respiratory disease is a subject of dispute. Some researchers suggest an association between WUPyV and KIPyV and respiratory symptoms (*1*,*3*,*5*), whereas others question the association between these viruses and disease (*7*,*8*). Here we report that WUPyV and KIPyV were the only viruses found in several samples, most of them originating from symptomatic episodes. Although these findings suggest a pathogenic role for both viruses, more extensive data are needed to establish their definite role in respiratory disease. In addition, we stress that we did not look for parainfluenza virus, human bocavirus, and coronavirus HKU1 in our study. We cannot exclude the fact that these viruses might be present in the samples in which only KIPyV or WUPyV was detected.

Because latent infections with subsequent asymptomatic reactivation are described as a feature of the polyomaviruses BK and JC (*12*), we were interested in the longitudinal course of WUPyV and KIPyV infections. Because most previous studies have a cross-sectional design, reinfections and persistence of WUPyV and KIPyV are usually missed. One study reported that WUPyV was detected in sequential samples of 2 immunocompromised patients for 6–8 weeks; 1 child was 16 months and the other 4 years of age (*5*). In the present study, positive episodes of WUPyV were interrupted by intervals during which no virus was detected in some children; however, the low viral load in these samples (high cycle threshold values) may have been below the detection limit in these negative intervals. In addition, we cannot exclude the possibility that poor collection techniques of samples with low viral loads resulted in failure to detect WUPyV.

We observed periods of successive positive samples for both WUPyV and KIPyV. These results might indicate that both viruses are able to persist in the respiratory tract. However, these positive samples could also represent new infections of WUPyV and KIPyV. Genetic analysis is needed to investigate whether this observed persistence is actual persistence or whether it represents new infections with different WUPyV and KIPyV subtypes. We conclude that WUPyV and KIPyV are frequently present in young children. Additional studies are needed to confirm the suggestion from this study that both viruses may be associated with respiratory disease.

## References

[1] Gaynor AM, Nissen MD, Whiley DM, Mackay IM, Lambert SB, Wu G, Identification of a novel polyomavirus from patients with acute respiratory tract infections. PLoS Pathog. 2007;3:e64. 10.1371/journal.ppat.0030064 17480120 PMC1864993

[2] Allander T, Andreasson K, Gupta S, Bjerkner A, Bogdanovic G, Persson MA, Identification of a third human polyomavirus. J Virol. 2007;81:4130–6. 10.1128/JVI.00028-07 17287263 PMC1866148

[3] Bialasiewicz S, Whiley DM, Lambert SB, Jacob K, Bletchly C, Wang D, Presence of the newly discovered human polyomaviruses KI and WU in Australian patients with acute respiratory tract infection. J Clin Virol. 2008;41:63–8. 10.1016/j.jcv.2007.11.001 18083616 PMC7108439

[4] Foulongne V, Brieu N, Jeziorski E, Chatain A, Rodiere M, Segondy M. KI and WU polyomaviruses in children, France. Emerg Infect Dis. 2008;14:523–5. 10.3201/eid1403.071206 18325286 PMC2570830

[5] Le BM, Demertzis LM, Wu G, Tibbets RJ, Buller R, Arens MQ, Clinical and epidemiologic characterization of WU polyomavirus infection, St. Louis, Missouri. Emerg Infect Dis. 2007;13:1936–8. 18258052 10.3201/eid1312.070977 PMC2876771

[6] Abed Y, Wang D, Boivin G. WU polyomavirus in children, Canada. Emerg Infect Dis. 2007;13:1939–41. 18258053 10.3201/eid1312.070909 PMC2876769

[7] Han TH, Chung JY, Koo JW, Kim SW, Hwang ES. WU polyomavirus in children with acute lower respiratory tract infections, South Korea. Emerg Infect Dis. 2007;13:1766–8. 18217567 10.3201/eid1311.070872 PMC2878209

[8] Norja P, Ubillos I, Templeton K, Simmonds P. No evidence for an association between infections with WU and KI polyomaviruses and respiratory disease. J Clin Virol. 2007;40:307–11. 10.1016/j.jcv.2007.09.008 17997354 PMC7172997

[9] van der Zalm MM, Uiterwaal CS, de Jong BM, Wilbrink B, van der Ent CK. Viral specimen collection by parents increases response rate in population-based virus studies. J Allergy Clin Immunol. 2006;117:955–6. 10.1016/j.jaci.2006.01.006 16630966

[10] Bialasiewicz S, Whiley DM, Lambert SB, Gould A, Nissen MD, Sloots TP. Development and evaluation of real-time PCR assays for the detection of the newly identified KI and WU polyomaviruses. J Clin Virol. 2007;40:9–14. 10.1016/j.jcv.2007.07.015 17714984 PMC7108442

[11] van Gageldonk-Lafeber AB, Heijnen ML, Bartelds AI, Peters MF, van der Plas SM, Wilbrink B. A case-control study of acute respiratory tract infection in general practice patients in The Netherlands. Clin Infect Dis. 2005;41:490–7. 10.1086/431982 16028157 PMC7107976

[12] Dorries K. Molecular biology and pathogenesis of human polyomavirus infections. Dev Biol Stand. 1998;94:71–9. 9776228

